# Life-course body size and perimenopausal mammographic parenchymal patterns in the MRC 1946 British birth cohort

**DOI:** 10.1038/sj.bjc.6601207

**Published:** 2003-08-26

**Authors:** V A McCormack, I dos Santos Silva, BL De Stavola, N Perry, S Vinnicombe, A J Swerdlow, R Hardy, D Kuh

**Affiliations:** 1 Department of Epidemiology and Population Health, London School of Hygiene and Tropical Medicine, London WC1E 7HT, UK; 2 Department of Diagnostic Imaging, St Bartholomew's Hospital, London EC1 7BE, UK; 3 Medical Research Council National Survey of Health and Development, Department of Epidemiology and Public Health, Royal Free & University College Medical School, 1-19 Torrington Place, London WC1E 6BT, UK

**Keywords:** mammographic parenchymal patterns, breast density, growth, life-course, body size

## Abstract

Dense mammographic parenchymal patterns are associated with an increased risk of breast cancer. Certain features of body size have been found to be associated with breast cancer risk, but less is known about their relation to breast density. We investigated the association of birth size, childhood growth and life-course changes in body size with Wolfe grade in 1298 perimenopausal women from a British cohort of women born in 1946. The cohort benefits from repeated measures of body size in childhood and adulthood. We obtained mammograms for 90% of women who at age 53 years reported having previously had a mammogram. We found no associations with birth weight or maximum attained height. Body mass index (BMI) at age 53 years and breast size were independently and inversely associated with Wolfe grade (*P*-value for trend <0.001 for both). Women who reached puberty later were at a greater odds of a higher Wolfe grade than women who had an earlier puberty (odds ratio associated with a 1 year delay in menarche 1.14, 95% CI: 1.01–1.27, adjusted for BMI and breast size at mammography). A higher BMI at any age during childhood or adult life was associated with a reduction in the odds of a higher Wolfe grade, after controlling for breast size and BMI at mammography, for example, standardised odds ratio for height at age 7 was 0.72 (95% CI: 0.64, 0.81). These findings reveal the importance of taking life-course changes in body size, and not just contemporaneous measures, into account when using mammographic density as an intermediate marker for risk of breast cancer.

Mammographic density has been found to be strongly and independently associated with an increased risk of subsequent breast cancer. Several epidemiological studies have reported the magnitude of this association to be about four- to six-fold comparing women with percent densities (percentage of the mammogram having a dense appearance) of 75% or greater to those with no dense areas (Byrne *et al*, 1995; Boyd *et al*, 1995), and about two- to four-fold when breast density was measured indirectly using the qualitative classification of breast parenchymal patterns proposed by Wolfe (DY/P2 *vs* P1/N1) (Gravelle *et al*, 1986). It has been estimated that having any area of dense mammographic appearance is responsible for 46% of all breast cancer cases in the US, with percent densities of 50% or more accounting for 28% of cases (Byrne *et al*, 1995). Comparisons of risk factor–breast density associations with corresponding established risk factor–breast cancer associations may shed light on the biological pathways through which risk factors operate. Such comparisons may also inform strategies by which this important modifiable risk factor and ultimately breast cancer risk might be reduced. Furthermore, the identification of factors that influence breast density, but not breast cancer risk, may reveal associations that need to be taken into account when using breast density as an intermediate marker for breast cancer risk.

Various markers of a woman's growth, such as birth weight (Michels *et al*, 1996; De Stavola *et al*, 2000; McCormack *et al*, 2003) and adult height (van den Brandt *et al*, 2000) have been shown to be associated with breast cancer risk, but less is known about their associations with mammographic density. To our knowledge, only one study (Ekbom *et al*, 1995) to date has investigated the potential role of perinatal characteristics and found a positive but nonsignificant association with birth weight. Inverse associations have been found between contemporaneous measures of adult adiposity and breast density (Brisson *et al*, 1984; Grove *et al*, 1985; Whitehead *et al*, 1985; De Stavola *et al*, 1990; Gram *et al*, 1997; Boyd *et al*, 1998; Sala *et al*, 1999), but associations with adult height have been less consistent (Brisson *et al*, 1984; Gram *et al*, 1997; Boyd *et al*, 1998; Sala *et al*, 1999). These studies were, however, limited to anthropometric measures relating to a single point in time, mostly at mammography, thus a detailed study of the effect of growth and life-course changes in body size could not be investigated. In this study, we had the opportunity to investigate Wolfe mammographic parenchymal patterns in relation to life-course changes in body size in a British cohort of women who have been followed since their births in 1946.

## MATERIALS AND METHODS

### Data sources

The MRC National Survey of Health and Development consists of a socially stratified sample of all single legitimate births in Britain during 3–9 March 1946 and initially included 2547 girls. Home visits to members of the cohort were made at ages 2, 4, 6, 7, 11, 15, 26, 36, 43 and 53 years, providing data on age at menarche, parity, age at first birth and social class, as well as height and weight (measured at all ages except 26 when it was self-reported). Ninety percent of women had complete height data on at least four of the six occasions analysed (i.e. ages 2, 4, 7, 11, 15 and one adult measure), with a similar percentage (89.7%) of completeness for BMI on at least six of the nine occasions (i.e. ages 2, 4, 7, 11,15, 26, 36, 43 and 53). Birth weight data, recorded to the nearest quarter of a pound, were obtained from hospital records within a few weeks of delivery and converted into grams. Data on the use of hormone replacement therapy (HRT) and menopausal status at the time of mammography, and, where appropriate, age at menopause were derived from information on menstrual and HRT histories obtained from annual questionnaires sent to the women from ages 47–54 years (Kuh and Hardy, in press). Women were considered retrospectively to be postmenopausal after 12 months of amenorrhea, perimenopausal if periods had stopped for between 3 and 12 months or had become more irregular in the preceding 12 months and premenopausal if still menstruating. Menopausal status could not be determined for women who had a hysterectomy or bilateral oophorectomy or who were taking HRT preparations that cause bleeding prior to inception of the menopause.

In the UK, all women aged 50–64 years are invited for 3-yearly mammographic screening as part of the NHS Breast Screening Programme. At a home visit in 1999 (when the women were aged 53 years), women were asked permission for us to obtain copies of their mammograms and details of the date(s) and clinic(s)/hospital(s) where they were undertaken. Copies of the mammograms (two views for each breast) taken when the women were closest to age 50 years were then requested from the relevant centres for all women who gave consent, providing a cross-sectional sample. The mammograms were jointly classified by two radiologists (NP and SV) using the Wolfe grade. This classification consists of four categories in order of increasing breast density: N1–predominantly fat, no ducts, only small amounts of dysplasia; P1–mainly fat, with ducts up to 25% of the breast; P2–ductal pattern in 25–75% of the breast; DY–dysplasia/fibrocystic change, sheet-like areas of higher density (Wolfe, 1976). Breast size was measured as the distance (to the nearest 0.1 cm) between the pectoralis muscle and nipple on the film. To assess within-observer agreement of the Wolfe grade, 110 randomly selected sets were reclassified by the same two radiologists several months later.

Of the original 2547 women in the cohort, no attempt was made at age 53 years to contact women who had previously refused to participate (12%), emigrated with no further contact (8%), or had died (8%). Of the 1848 eligible women, 1600 (87%) participated and of these, 1494 (93%) reported having had a mammogram, of whom almost all (*n*=1471) consented access to their mammograms.

### Statistical methods

We used cumulative logit models for ordinal outcomes (Armstrong and Sloan, 1989) to study factors that might influence Wolfe grade. These models assume that the effect of each explanatory variable on any dichotomy of the ordinal outcome is constant and can be summarised by a common odds ratio. In our study, this corresponds to assuming a common odds ratio independently of whether Wolfe grade is dichotomised as (P1, P2, DY) *vs* (N1); (P2, DY) *vs* (N1, P1); or (DY) *vs* (N1, P1, P2). This assumption was tested via an approximate log likelihood ratio test that compares the cumulative logit model with a polytomous logistic model (Anderson, 1984) in which three separate logistic regressions are performed. Hereafter, the reported common odds ratios are referred to as ‘odds ratios for a higher Wolfe grade’ (with N1 as the lowest and DY as the highest grade), and they should be regarded as prevalence odds ratios as the Wolfe grade information was obtained cross-sectionally.

The main exposures of interest were birth weight, age at menarche and body size in infancy, childhood and adulthood, as measured by height and, as a measure of adiposity, body mass index (BMI) (calculated as weight (kg) × height^−2^ (m^−2^)) at ages 2, 4, 7, 11, 15, 26, 36, 43 and 53 years. Age-specific attained height and BMI were analysed as continuous variables. Average height and BMI velocities between consecutive ages, calculated as the change in height and BMI respectively divided by the time interval (in years, to the nearest month) between the two visits, were also analysed as continuous variables. These velocities capture growth during particular age intervals, whereas attained heights or BMIs reflect cumulative exposure up to a particular age. We calculated standardised heights and BMIs for each age and standardised velocities for each interval so that a standardised measure of effect could be compared across different ages/intervals.

To overcome the loss of subjects from multivariable analyses that included several height/BMI measurements simultaneously, we imputed missing growth data. Firstly, we modelled the height and BMI trajectories (separately) using random effects growth models. Height was modelled as a linear function of age between ages 2–4 and 4–7 years and a quadratic function from age 7 to adulthood. Body mass index was modelled as a quadratic function of age during ages 2–7 and 7–15 years and a linear function between ages 15–26, 26–36, 36–43 and 43–53 years. In both growth models, all effects were random, except for the quadratic terms, and the random effects parameters depended on the final Wolfe grade. Imputed values for missing heights/BMIs were generated by firstly taking a random draw of the error terms, then taking a random draw of the growth parameters and using these to predict imputed values for the missing growth data. This procedure was repeated five times to create five datasets with complete data (observed data plus those imputed if missing). Complete case analyses were then carried out on each dataset. Summary odds ratios (geometric means of the five odds ratios) and their 95% confidence intervals were calculated as in Schafer (1999).

Age at first birth, parity, years since last birth, HRT use, menopausal status and years postmenopausal were considered as potential confounders. We adjusted for breast size and BMI at age 53 years in all analyses in order to investigate associations with changes in body size independent of body size at mammography, as obesity at mammography is known to be a strong confounder of the mammographic parenchymal pattern (Bartow *et al*, 1995; Salminen *et al*, 1998; Sala *et al*, 1999) and needs to be taken into account when separate measures of the areas of dense and nondense breast are not available.

## RESULTS

We successfully obtained copies of mammograms for 1319 of the 1471 women who gave consent (90% tracing rate), the large majority (1249 (95%)) from NHS breast screening centres. Films were not obtained for the remaining women due to insufficient information to contact hospitals/clinics (37 in UK, 12 overseas), logistic difficulties in certain centres (57) and other problems (45). We obtained both cranio-caudal and oblique views of each breast for 89% of received sets. In all, 21 women with a diagnosis of breast cancer prior to mammography were excluded from analyses, as treatment for breast cancer may influence the outcome. Thus, the present analyses are based on 1298 women.

Eighty-two percent (90 out of 110) of mammograms that were read twice had the same Wolfe grade on both occasions and the remaining pairs differed by only one grade. The weighted *κ* statistic (to account for the ordered nature of the categories) was 0.79 (*P*<0.001) and using the binary classification (combining DY with P2 and P1 with N1) was 0.83 (*P*<0.001). Table 1

**Table 1:** Characteristics of the study population by Wolfe grade and associated odds ratios for a higher Wolfe grade^a^

	**Wolfe grade**					
	**N1**	**P1**	**P2**	**DY**	**Odds ratio**^a^ ***(P for linear trend where appropriate)***	**95% confidence interval**
(*N* (column %) unless stated otherwise)						
*N*^b^ (row %)	175 (13.5)	358 (27.6)	682 (52.5)	83 (6.4)	—	—
Age at mammogram (years)	51.5 (1.0)	51.5 (1.0)	51.6 (1.1)	51.4 (1.2)	1.06^c^	0.96, 1.16
(mean (s.d.))					(*P*=0.24)	
Age at menarche (years)	12.7 (1.3)	12.9 (1.2)	13.1 (1.3)	13.4 (1.2)	1.20^c^	1.10, 1.30
(mean (s.d.))					(*P*<0.001)	

Age at first birth (years)^d^						
<20	18 (11.5)	49 (15.3)	69 (11.9)	3 (4.3)	1	—
20–24	83 (52.9)	146 (45.6)	265 (45.5)	26 (37.1)	1.12	0.80, 1.58
25–29	41 (26.1)	91 (28.4)	186 (32.0)	31 (44.3)	1.56	1.09, 2.25
≥30	15 (9.6)	34 (10.6)	62 (10.7)	10 (14.3)	1.41	0.89, 2.22
					(*P*^d^=0.02)	

Nulliparous Parity^d^	15 (8.7)	34 (9.6)	89 (13.3)	10(12.5)	1.19	0.77, 1.85
1	19 (12.3)	41 (13.1)	83 (14.4)	12 (17.6)	1	—
2	70 (45.2)	161 (51.3)	308 (53.6)	39 (57.4)	0.94	0.67, 1.33
3	43 (27.7)	73 (23.2)	148 (25.7)	14 (20.6)	0.82	0.56, 1.20
4+	23 (14.8)	39 (12.4)	36 (6.3)	3 (4.4)	0.41	0.26, 0.66
					(*P*^d^=0.001)	

Menopausal status						
Post	47 (26.9)	89 (24.9)	158 (23.2)	13 (15.7)	1	—
Peri	28 (16.0)	55 (15.4)	119 (17.5)	16 (19.3)	1.33	0.95, 1.85
Pre	22 (12.6)	57 (15.9)	127 (18.6)	18 (21.7)	1.53	1.10, 2.12
Hysterectomy	38 (21.7)	70 (19.6)	123 (18.0)	13 (15.7)	1.02	0.74, 1.40
NK as on HRT	29 (16.6)	74 (20.7)	118 (17.3)	21 (25.3)	1.21	0.88, 1.67
NK, other	11 (6.3)	13 (3.6)	37 (5.4)	2 (2.4)	—	—

HRT use						
Never users	102 (58.3)	191 (53.4)	370 (54.3)	42 (50.6)	1	—
Ex-users	29 (16.6)	56 (15.6)	108 (15.8)	11 (13.3)	0.99	0.74, 1.33
Current users	25 (14.3)	63 (17.6)	93 (13.6)	18 (21.7)	1.02	0.75, 1.37
Other^e^	19 (10.9)	48 (13.4)	108 (15.8)	12 (14.5)	1.27	0.93, 1.74

Smoking status^f^						
Never smokers	80 (45.7)	168 (46.9)	333 (48.8)	40 (48.2)	1	—
Ex-smokers	54 (30.9)	101 (28.2)	200 (29.3)	20 (24.1)	0.92	0.72, 1.17
Current smokers	39 (22.3)	82 (22.9)	135 (19.8)	17 (20.5)	0.86	0.66, 1.12
NK	2(1.1)	7 (2.0)	14 (2.1)	6 (7.2)	—	—

Social class						
Manual	94 (53.7)	217 (60.6)	440 (64.5)	60 (72.3)	1	—
Nonmanual	53 (30.3)	90 (25.1)	146 (21.4)	8 (9.6)	1.60	1.25, 2.06
NK	28 (16.0)	51 (14.3)	96 (14.1)	15 (18.1)	—	—

a Common odds ratio for three binary outcomes: DY *vs* P2/P1/N1, DY/P2 *vs* P1/N1 and DY/P2/P1 *vs* N1.

b *N* varies due to missing data.

c Odds ratio associated with a 1 year increase in the explanatory variable.

d Parous women only.

e Women who commenced HRT before mammography, but date of end of HRT episode not known.

f Reported at age 53 years.NK=not known.

shows the distribution of breast cancer risk factors by Wolfe grade and their associated odds ratios for a higher Wolfe grade. Just over half (53%) of the women in the study had a P2 grade and 6% a DY grade. The mean age of women at mammography was similar in each Wolfe category. On average, higher Wolfe grades were significantly associated with a later age at menarche, a later age at first birth, low parity or nulliparity, and with being pre-/perimenopausal. Women in nonmanual social classes were also more likely to have a higher Wolfe grade relative to women in manual social classes. There was no association between Wolfe grade and smoking status or HRT use. The distribution of reproductive factors and anthropometric measures did not differ between women for whom we did and did not obtain a mammogram (results not shown).

**Figure 1:**
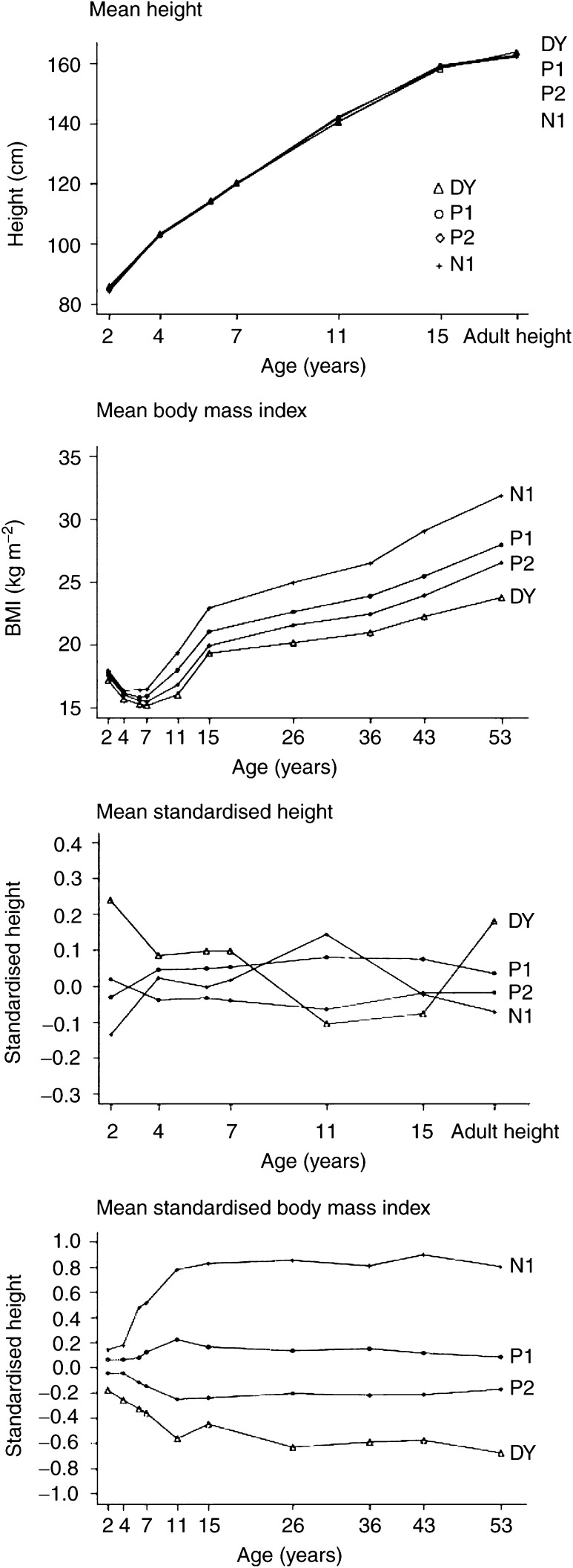
Mean age-specific crude and standardised heights and BMIs by Wolfe grade

The mean BMI at each age was lower for higher Wolfe grades and the differences between Wolfe-specific means increased from age 11 years onwards (Figure 1

). Wolfe grade-specific height profiles were less distinct from each other. Mean height among women with a DY grade was slightly higher up to age 7 years and again in adulthood, but was lower during adolescence. Breast size and BMI at age 53 years had strong inverse associations with Wolfe grade (Table 2

**Table 2:** Odds ratios and 95% confidence intervals (95% CI) for a higher Wolfe grade^a^ associated with breast size and BMI at age 53

	**Univariable analysis**		**Bivariable analysis^b^**	
	**Odds ratio^a^**	**95% CI**	**Odds ratio^a^**	**95% CI**
Breast size (cm)				
3–6	4.07	2.50, 6.62	2.62	1.54, 4.45
7–8	1.76	1.31, 2.38	1.45	1.06, 1.99
9–10	1		1	
11–12	0.66	0.49, 0.88	0.85	0.63, 1.15
≥13	0.40	0.30, 0.54	0.75	0.52, 1.06
			(*P*^c^<0.001)	

Fifths of BMI at age 53 (kg m^−2^)				
<23.1	1		1	
23.1–25.15	0.67	0.47, 0.97	0.79	0.55, 1.14
25.16–27.57	0.45	0.32, 0.64	0.57	0.39, 0.82
27.58–31.39	0.32	0.23, 0.46	0.45	0.31, 0.65
≥31.4	0.13	0.09, 0.19	0.21	0.14, 0.32
			(*P*^c^<0.001)	

a Common odds ratio for three binary outcomes: DY *vs* P2/P1/N1, DY/P2 *vs* P1/N1 and DY/P2/P1 *vs* N1.

b Breast size adjusted for fifths of BMI at age 53 years and *vice versa*.

c *P*-value for linear trend.

), that is, women with a lower BMI and smaller breast size had on average more dense Wolfe patterns. The univariable odds ratios were attenuated when each of these variables was controlled for the other, due to their positive correlation; however, both remained independently associated with Wolfe grade and are thus controlled for in subsequent analyses.

We found no evidence of an association between birth weight and Wolfe grade (Table 3

**Table 3:** Odds ratios, 95% confidence intervals (CI) and *P*-values for a higher Wolfe grade^a^ associated with an s.d. increase in birth weight, age at menarche, age-specific heights and interval-specific height velocities

	**Observed data only**				**Including imputed values where missing and controlling for:**							
			**Crude**		**Crude (*N*=1263)**		**Age at mammography, BMI at age 53 years and breast size (*N*=1253)**			**All other height components, age at mammography, breast size, BMI age 53 (*N*=1**253)		
	***n***^b^	**Mean (s.d.)**	**Odds ratio^a^**	**95% CI**	**Odds ratio^a^**	**95% CI**	**Odds ratio^a^**	**95% CI**	***P***-value	**Odds ratio^a^**	**95% CI**	***P***-value
Birth weight (g)												
	1294	3318 (468)	1.03	0.93, 1.14	—	—	1.03^c^	0.92, 1.15	0.59	—	—	—
Age at menarche (years)												
	1214	13.0 (1.3)	1.26	1.13, 1.40	—	—	1.14^c^	1.01, 1.27	0.03	—	—	—
Height (cm) at age												
2	1033	84.7 (4.6)	1.15	1.02, 1.29	1.09	0.98, 1.21	1.13	1.01, 1.26	0.03	1.07	0.94, 1.22	0.31
4	1123	102.9 (5.0)	0.96	0.86, 1.08	0.96	0.86, 1.07	0.94	0.84, 1.05	0.25	—	—	—
7	1122	119.8 (5.5)	0.97	0.86, 1.08	0.94	0.85, 1.05	0.93	0.84, 1.04	0.22	—	—	—
11	1090	141.2 (7.0)	0.85	0.76, 0.95	0.88	0.79, 0.97	0.89	0.80, 1.00	0.04	—	—	—
15	1002	158.7 (6.2)	0.95	0.84, 1.07	0.99	0.89, 1.10	0.96	0.86, 1.07	0.47	—	—	—
36	1174	162.5 (6.3)	1.07	0.96, 1.20	1.08	0.97, 1.20	1.03	0.93, 1.15	0.55	—	—	—
Height velocity (cm/year) during ages												
2–4	966	7.94 (2.2)	0.87	0.77, 0.98	0.87	0.78, 0.97	0.82	0.74, 0.92	<0.001	0.85	0.76, 0.96	0.01
4–7	1015	6.10 (1.4)	0.97	0.86, 1.08	0.98	0.88, 1.09	1.00	0.90, 1.12	0.97	0.97	0.86, 1.09	0.60
7–11	1030	5.64 (1.1)	0.85	0.76, 0.96	0.88	0.80, 0.98	0.92	0.82, 1.02	0.13	1.00	0.86, 1.15	0.96
11–15	950	4.77 (1.3)	1.19	1.05, 1.34	1.18	1.06, 1.31	1.11	1.00, 1.24	0.06	1.11	0.99, 1.24	0.08
15–adulthood	927	1.07 (1.0)	1.22	1.08, 1.38	1.18	1.06, 1.32	1.15	1.03, 1.29	0.01	1.16	1.03, 1.29	0.01

a Common odds ratio associated with 1 s.d. increase in explanatory variable for three binary outcomes: (DY *vs* P2,P1,N1), (DY,P2 *vs* P1,N1) and (DY,P2,P1 *vs* N1).

b Number of women with nonmissing data for analyses on observed data only (excluding imputed values).

c Observed data only, that is, no imputed values.

). By contrast, women who were taller at age 2 years had an increased odds of a greater Wolfe grade. In univariable analyses, women who had greater height velocities in early childhood (2–4 years), later childhood (7–11 years) or were taller at age 11 years had a statistically significant lower odds of a greater Wolfe grade, whereas those with greater height velocities after age 11 years were at an increased odds. These associations were not confounded by breast size or BMI at mammography. Controlling for all components of the height growth trajectory (height at age 2 and height velocities thereafter), breast size and BMI at age 53 years simultaneously, height velocities during two intervals remained statistically significantly associated with Wolfe grade: greater height velocity from age 2 to 4 years was associated with a reduced odds, whereas greater height gain beyond age 15 years was associated with an increased odds of a greater Wolfe grade.

A later menarche was associated with an increased odds of a greater Wolfe grade, which persisted after controlling for BMI at age 53 years and breast size. Evidence that women with higher Wolfe grades had, on average, later puberty was found with other pubertal indicators in this cohort. For example, the percentage of women who had signs of breast development by age 11 years were 56.5, 51.3, 42.0 and 32.9% for women with Wolfe grades N1, P1, P2 and DY, respectively.

We found no evidence of an association between measured adult height at age 36 years and Wolfe grade (odds ratio for 6 cm increase: 1.06, 95% CI: 0.95, 1.19). However, in the same group of women, measured adult height at age 53 years had a statistically significant association with Wolfe grade (corresponding odds ratio 1.15, 95% CI: 1.04, 1.28). This apparent discrepancy was explained by greater height reduction, on average, in women who subsequently had lower Wolfe grades, for example, mean (95% CI) height loss between these ages was 1.29 cm (1.07, 1.50) and 0.79 cm (0.66, 0.92) in women with N1 and P2 Wolfe grades, respectively.

We found strong and highly statistically significant crude inverse associations with BMI at every age (Table 4

**Table 4:** Odds ratios, 95% confidence intervals (CI) and *P*-values for a greater Wolfe grade^a^ associated with an s.d. increase in age-specific BMI and interval-specific BMI velocities

	**Original observed data only**				**Including imputed values where missing and controlling for**							
			**Crude**		**Crude (*N*=1263)**		**Age at mammography, BMI at age 53 years and breast size (N=1287)**			**All other BMI components, age at mammography and breast size (N=1287)**		
	***n***^b^	**Mean (s.d.)**	**Odds ratio^a^**	**95% CI**	**Odds ratio^a^**	**95% CI**	**Odds ratio^a^**	**95% CI**	***P***-value	**Odds ratio^a^**	**95% CI**	***P***-value
BMI (kg m^−2^) at age												
2	994	17.6 (2.4)	0.85	0.76, 0.96	0.89	0.80, 0.99	0.88	0.79, 0.98	0.02	0.54	0.43, 0.71	<0.001
4	1093	16.1 (1.6)	0.83	0.74, 0.92	0.83	0.75, 0.92	0.89	0.80, 0.99	0.03	—	—	—
7	1074	15.7 (1.5)	0.63	0.56, 0.71	0.62	0.56, 0.69	0.72	0.64, 0.80	<0.001	—	—	—
11	1079	17.4 (2.5)	0.46	0.41, 0.52	0.46	0.41, 0.52	0.56	0.49, 0.63	<0.001	—	—	—
15	989	20.6 (2.8)	0.47	0.41, 0.53	0.45	0.40, 0.51	0.56	0.49, 0.64	<0.001	—	—	—
26	1129	22.2 (3.2)	0.45	0.39, 0.51	0.45	0.39, 0.51	0.62	0.53, 0.73	<0.001	—	—	—
36	1168	23.3 (3.9)	0.47	0.41, 0.53	0.47	0.41, 0.53	0.64	0.54, 0.78	<0.001	—	—	—
43	1209	24.9 (4.6)	0.45	0.40, 0.51	0.45	0.40, 0.51	0.54	0.43, 0.68	<0.001	—	—	—
53	1255	27.5 (5.5)	0.49	0.44, 0.56	0.49	0.44, 0.56	—	—	—	—	—	—
BMI velocity ((kg m^−2^) year^−1^) during ages												
2–4	911	−0.67 (1.1)	1.02	0.90, 1.15	0.99	0.89, 1.10	1.05	0.94, 1.17	0.41	0.56	0.49, 0.64	<0.001
4–7	950	−0.13 (0.6)	0.81	0.71, 0.91	0.79	0.71, 0.88	0.85	0.76, 0.95	0.004	0.62	0.54, 0.71	<0.001
7–11	975	0.45 (0.5)	0.53	0.46, 0.60	0.54	0.48, 0.60	0.63	0.56, 0.71	<0.001	0.50	0.43, 0.59	<0.001
11–15	929	0.86 (0.5)	0.89	0.78, 1.00	0.89	0.80, 0.98	0.98	0.87, 1.09	0.69	0.72	0.63, 0.81	<0.001
15–26	904	0.15 (0.2)	0.85	0.74, 0.96	0.88	0.78, 0.99	1.11	0.98, 1.26	0.11	0.72	0.63, 0.83	<0.001
26–36	1054	0.10 (0.3)	0.82	0.73, 0.92	0.82	0.73, 0.92	1.13	0.99, 1.29	0.06	0.86	0.75, 0.99	0.03
36–43	1119	0.23 (0.3)	0.78	0.70, 0.88	0.78	0.70, 0.88	0.99	0.87, 1.12	0.85	0.85	0.75, 0.98	0.02
43–53	1188	0.26 (0.3)	0.90	0.80, 1.00	0.90	0.80, 1.00	1.46	1.27, 1.68	<0.001	0.94	0.82, 1.07	0.33

a Common odds ratio for three binary outcomes: DY *vs* P2/P1/N1, DY/P2 *vs* P1/N1 and DY/P2/P1 *vs* N1 associated with 1 s.d. increase in explanatory variable.

b Number of women with nonmissing data for analyses on observed data only (excluding imputed values).

), findings that were partially confounded by BMI at age 53 years and breast size. After controlling for these measures, however, an s.d. increase in adult BMI at any previous age remained associated with approximately a 40% reduction in the odds of a higher Wolfe grade. Similarly, BMI velocities from age 4 years onwards were inversely associated with greater Wolfe grades in the crude analysis. Comparing women of similar BMI at age 53 years and breast size, greater rates of increase in BMI during preadolescent years, considered one at a time, remained significantly associated with a lower Wolfe grade, whereas a greater increase in BMI during ages 43–53 years (which reflects a lower BMI at age 43 years when BMI at age 53 years is held constant) was associated with an increased odds. Once breast size at mammography and all other components of a woman's BMI trajectory up to age 53 years were controlled for, an increase in BMI during any period up to age 43 years was associated with a reduced odds of a greater Wolfe grade, particularly during preadolescent years (7–11 years). Conditional on a woman's BMI trajectory up to age 43 years and breast size at mammography, changes in BMI thereafter were not associated with Wolfe grade.

Odds ratios in analyses that were restricted to the observed data were very similar to those that included imputed values that where missing (first two sets of odds ratios in Table 3 and 4). Further adjustment for the potential confounders (age at first birth, years since last birth, parity, menopausal status, years postmenopausal and HRT use) did not alter the results for height and BMI.

## DISCUSSION

### Advantages and limitations

Advantages of this study include the representativeness of the participants (Wadsworth *et al*, in press) and a high tracing rate of mammograms (91%). However, we were less likely to include mammograms taken privately or overseas or those taken for diagnostic (symptomatic) rather than screening reasons. Diagnostic mammograms are more likely to be of higher densities, hence there may be a small underestimate of P2 or DY proportions. We avoided the loss of subjects in multivariable analyses by modelling the growth curve and imputing missing values. We used BMI as a measure of weight relative to height at all ages for reasons of consistency and familiarity; however, this index is not independent of height at younger ages (Cole, 1986). Furthermore, we did not have BMI exactly at the time of mammography–the closest measurements at age 53 years were taken a mean of 2.1 years (90% reference range: 0.0–3.6 years) after mammography.

The Wolfe grade is not only a measure of breast density, but also includes other radiological features of the breast. Less subjective and possibly more informative outcomes would have been a quantitative measure of breast density and separate measures of the areas of dense and nondense breast tissue. These measures were not available to us at the time of publication and require further study. We took the ordered nature of the outcome variable into consideration by using a cumulative logit model that is more appealing than a binary logistic model (P2/DY *vs* N1/P1), commonly used to analyse such data, as the latter has reduced statistical power and disregards valuable information by combining categories (Armstrong and Sloan, 1989). We found very good within-observer agreement in a subsample. Differential error in the assessment of mammograms was avoided since reading was done without knowledge of a woman's risk factors, so any misclassification is likely to be nondifferential, which would result in the possible attenuation of effects. The Wolfe grade data were obtained cross-sectionally, so we were unable to investigate the effect of changes in body size on changes in density.

### Main findings

The strong inverse association of breast size with Wolfe grade is consistent with previous findings (Brisson *et al*, 1984; Salminen *et al*, 1998; Sala *et al*, 1999). Thus, despite measurement error due to varying degrees of breast compression during mammography, this crude measure of breast size (pectoralis muscle to nipple distance) is useful when no others are available.

We found no evidence of an association between birth weight and Wolfe grade, consistent with the small positive but nonsignificant association found in the only other study that has investigated *in utero* influences (Ekbom *et al*, 1995).

Differential height reduction between ages 36 and 53 years, which was greatest for women who subsequently had less-dense Wolfe grades, explained the differences between the null association with height at age 36 years and the positive association at age 53 years. Height loss between these ages was comparable to changes found in women in the Baltimore Longitudinal Study of Aging (Sorkin *et al*, 1999). Thus, the age to which ‘adult height’ refers and whether it was measured or self-reported may explain the conflicting previous findings (Brisson *et al*, 1984; Gram *et al*, 1997; Boyd *et al*, 1998; Sala *et al*, 1999). This observation may reveal the role of common determinants of both breast density and bone mineral density/height loss, such as changes in oestrogen levels.

We found that early maturers (characterised by a greater height velocity up to age 11 years, greater height at this age and an earlier menarche) were at a reduced odds of a higher Wolfe grade compared to later maturers. In view of the association of early maturation with high adult BMI (Parsons *et al*, 1999) and the strong inverse association of the latter with breast density, we expected these associations in the crude analysis. The association with timing of maturation persisted, however, even after controlling for BMI at mammography.

Our results for adult BMI are in agreement with previously reported associations of adiposity and breast density at mammography (Brisson *et al*, 1984; Whitehead *et al*, 1985; Bartow *et al*, 1995; Gram *et al*, 1997; Boyd *et al*, 1998; Sala *et al*, 1999). As several studies have illustrated a tracking of obesity from childhood to adult life (Parsons *et al*, 1999), we envisaged the observed crude inverse association with childhood BMI. The associations with BMI and BMI changes during childhood remained, however, after controlling for breast size and adult BMI. Comparing women of similar breast size and BMI at mammography, women who had a high BMI throughout life were at lower odds of a higher density Wolfe grade than women who put on weight more recently.

### Determinants of breast density and their relation to breast cancer risk

A comparison of the determinants of breast density, and their mechanisms, with their corresponding associations with breast cancer risk may provide useful insights into ways in which breast density and ultimately breast cancer risk may be reduced. Breast density is a measure of the relative proportions of radiolucent adipose tissue and radiodense epithelial (parenchyma) and connective (stroma) tissue. Radiodense tissue possibly reflects the number of cells at risk of carcinogenesis as it is in the ducts and lobules of the epithelium where the vast majority of breast cancers originate. In support of this mechanism, Brisson *et al* (1984) found that the percentage of the breast with nodular densities was shown to have a stronger association with breast cancer risk compared to Wolfe grade or percentage of breast with homogeneous densities. Dense mammographic patterns have been associated with changes of the parenchymal tissue such as epithelial hyperplasia and proliferation of the breast stroma where growth factors that influence the breast epithelium are produced (Oza and Boyd, 1993).

The strong associations of adiposity and body size with breast density are more likely to be driven by associations with the area of radiolucent rather than the area of dense breast tissue, as other studies have found positive associations between weight/BMI and the area of nondense tissue and less strong, but inverse, associations with the area of dense tissue (Boyd *et al*, 1998). Associations of body size with the area of dense tissue or with features of the parenchymal pattern that may reflect breast cancer risk are likely to be dwarfed by these. Associations of adiposity with breast cancer risk are, on the contrary, of much smaller magnitude. Adiposity has an inverse association with breast cancer risk at premenopausal ages (Willett *et al*, 1985) and a positive association at postmenopausal ages (Hunter and Willett, 1993). Findings from the present cohort are also consistent with an association of BMI with breast cancer at premenopausal ages (hazard ratio 0.76, 95% CI: 0.54–1.10, for 1 s.d. increase in BMI at age 36 years); the women in the cohort are still too young to allow analysis at postmenopausal ages. Endogenous oestrogen production in fatty tissue has been suggested as a possible mechanism through which adiposity affects breast cancer risk in postmenopausal women in whom ovarian oestrogen production has ceased (Pike *et al*, 1993).

Similar to body size, the observed associations of age at menarche and breast size with Wolfe grade are more likely to predominantly reflect associations with nondense breast tissue. Earlier menarche was associated with a decreased odds of a higher Wolfe grade. In contrast, and similarly to other studies (Hsieh *et al*, 1990), early menarche was associated with increased breast cancer risk in this cohort (odds ratio 1.43, 95% CI: 0.73–2.80, comparing women who had menarche under 12.5 years to those whose menarche was 13.5 years or over). Increasing breast size was strongly associated with a decreased odds of a higher Wolfe grade, but its association with breast cancer risk has not been consistent across studies (Hsieh and Trichopoulos, 1991; Byrne *et al*, 1995). Taller women have increased rates of breast cancer (van den Brandt *et al*, 2000), and although a similar association was found for breast cancer in this cohort (hazard ratio 1.37, 95% CI: 1.02–1.84 per s.d. increase in height at age 36 years), we did not observe a similar association with breast density. Similarly, the null findings for birth weight with Wolfe grade in the present analysis are in contrast to the positive associations found in this cohort (De Stavola *et al*, 2000) and other studies (Michels *et al*, 2002; McCormack *et al*, 2003). These contrasting associations of age at menarche, breast size, birth weight and adult height with breast density and breast cancer risk are suggestive of different biological pathways through which these pairs of associations operate.

The complex and long-term associations of the timing of maturation and life-course changes in body size with Wolfe grade have implications for studies that use breast density as an intermediate marker of breast cancer risk. Our findings suggest that life-course changes in body size, and not just adiposity at mammography, need to be taken into account to avoid spurious findings for exposures that are associated with these factors. The findings also imply that the association of Wolfe patterns and breast cancer risk previously reported may have been underestimated as they only took into account adiposity at the time of the mammography. These findings further emphasise the need to identify measures of mammographic features that are not so strongly influenced by adiposity (Boyd *et al*, 1998).

In summary, we have found that mammographic parenchymal patterns as measured by the Wolfe grade are not only strongly associated with contemporaneous adiposity and breast size, but also have strong complex associations with the trajectory of growth and body size throughout the life-course. It remains to investigate whether these associations differ for the areas of dense and nondense tissue as these associations may have implications for using these measures of breast density as intermediate markers of breast cancer risk.

## References

[bib1] Anderson JA (1984) Regression and ordered categorical variables. JR Stat Soc B 1: 1–30

[bib2] Armstrong B, Sloan M (1989) Ordinal regression models for epidemiologic data. Am J Epidemiol 129: 191–204 2910061 10.1093/oxfordjournals.aje.a115109

[bib3] Bartow SA, Pathak DR, Mettler FA, Key CR, Pike MC (1995) Breast mammographic pattern: a concatenation of confounding and breast cancer risk factors. Am J Epidemiol 142: 813–819 7572957 10.1093/oxfordjournals.aje.a117720

[bib4] Boyd NF, Byng JW, Jong RA, Fishell EK, Little LE, Miller AB, Lockwood GA, Tritchler DL, Yaffe MJ (1995) Quantitative classification of mammographic densities and breast cancer risk: results from the Canadian National Breast Screening Study. J Natl Cancer Inst 87: 670–675 7752271 10.1093/jnci/87.9.670

[bib5] Boyd NF, Lockwood GA, Byng JW, Little LE, Yaffe MJ, Tritchler DL (1998) The relationship of anthropometric measures to radiological features of the breast in premenopausal women. Br J Cancer 78: 1233–1238 9820186 10.1038/bjc.1998.660 PMC2063010

[bib7] Brisson J, Morrison AS, Kopans DB, Sadowsky NL, Kalisher L, Twaddle JA, Meyer JE, Henschke CI, Cole P (1984) Height and weight, mammographic features of breast tissue, and breast cancer risk. Am J Epidemiol 119: 371–381 6702813 10.1093/oxfordjournals.aje.a113755

[bib9] Byrne C, Schairer C, Wolfe J, Parekh N, Salane M, Brinton LA, Hoover R, Haile R (1995) Mammographic features and breast cancer risk: effects with time, age and menopause status. J Natl Cancer Inst 87: 1622–1629 7563205 10.1093/jnci/87.21.1622

[bib10] Cole TJ (1986) Weight/height^p^ compared to weight/height^2^ for assessing adiposity in childhood: influence of age and bone age on p during puberty. Ann Hum Biol 13: 433–451 3800308 10.1080/03014468600008621

[bib11] De Stavola BL, Gravelle IH, Wang DY, Allen DS, Bulbrook RD, Fentiman IS, Hayward JL, Chaudary MC (1990) Relationship of mammographic parenchymal patterns with breast cancer risk factors and risk of breast cancer in a prospective study. Int J Epidemiol 19: 247–254 2376431 10.1093/ije/19.2.247

[bib12] De Stavola BL, Hardy R, Kuh D, dos Santos Silva I, Wadsworth M, Swerdlow AJ (2000) Birthweight, childhood growth and risk of breast cancer in a British cohort. Br J Cancer 83: 964–968 10970703 10.1054/bjoc.2000.1370 PMC2374673

[bib13] Ekbom A, Thurfjell E, Hsieh CC, Trichopoulos D, Adami HO (1995) Perinatal characteristics and adult mammographic patterns. Int J Cancer 61: 177–180 7705944 10.1002/ijc.2910610206

[bib14] Gravelle IH, Bulstrode JC, Bulbrook RD, Wang DY, Allen D, Hayward JL (1986) A prospective study of mammographic parenchymal patterns and risk of breast cancer. Br J Radiol 59: 487–491 3708251 10.1259/0007-1285-59-701-487

[bib15] Gram IT, Funkhouser E, Tabar L (1997) Anthropometric indices in relation to mammographic patterns among peri-menopausal women. Int J Cancer 73: 323–326 9359476 10.1002/(sici)1097-0215(19971104)73:3<323::aid-ijc3>3.0.co;2-1

[bib16] Grove JS, Goodman MJ, Gilbert Jr FI, Mi MP (1985) Factors associated with mammographic pattern. Br J Radiol 58: 21–25 4063637 10.1259/0007-1285-58-685-21

[bib17] Hsieh CC, Trichopoulos D (1991) Breast size, handedness and breast cancer risk. Eur J Cancer 27: 131–135 1827274 10.1016/0277-5379(91)90469-t

[bib18] Hsieh CC, Trichopoulos D, Katsouyanni K, Yuasa S (1990) Age at menarche, age at menopause, height and obesity as risk factors for breast cancer: associations and interactions in an international case-control study. Int J Cancer 46: 796–800 2228308 10.1002/ijc.2910460508

[bib19] Hunter DJ, Willett WC (1993) Diet, body size and breast cancer. Epidemiol Rev 15: 110–132 8405195 10.1093/oxfordjournals.epirev.a036096

[bib20] Kuh D, Hardy R (2003) Women's health in midlife: findings from a British birth cohort study. J Br Menopause Soc 9: 55–60 10.1258/136218003100322206 12844426

[bib21] McCormack VA, dos Santos Silva I, De Stavola BL, Mohsen R, Leon DA, Lithell HO (2003) Fetal growth and subsequent risk of breast cancer: results from long term follow up of Swedish cohort. Br Mea J 326: 248–251 10.1136/bmj.326.7383.248 PMC140759 12560272

[bib22] Michels KB, Trichopoulos D, Robins JM, Rosner BA, Manson JE, Hunter DJ, Colditz GA, Hankinson SE, Speizer FE, Willett WC (1996) Birthweight as a risk factor for breast cancer. Lancet 348: 1542–1546 8950880 10.1016/S0140-6736(96)03102-9

[bib23] Oza AM, Boyd NF (1993) Mammographic parenchymal patterns: a marker of breast cancer risk. Epi Rev 5: 196–208 10.1093/oxfordjournals.epirev.a036105 8405204

[bib24] Parsons RJ, Power C, Logan S, Summerbell CD (1999) Childhood predictors of adult obesity: a systematic review. Int J Obesity 23(Suppl 8): S1–S107 10641588

[bib25] Pike MC, Spicer DV, Dahmoush L, Press MF (1993) Estrogens, progestogens, normal breast cell proliferation, and breast cancer risk. Epidemiol Rev 15: 17–35 8405201 10.1093/oxfordjournals.epirev.a036102

[bib26] Sala E, Warren R, McCann J, Duffy S, Luben R, Day N (1999) High-risk mammographic parenchymal patterns and anthropometric measures: a case–control study. Br J Cancer 81: 1257–1261 10584891 10.1038/sj.bjc.6690838 PMC2374338

[bib27] Salminen T, Hakama M, Meikkila M, Saarenmaa I (1998) Favorable change in mammographic parenchymal patterns and breast cancer risk factors. Int J Cancer 78: 410–414 9797126 10.1002/(sici)1097-0215(19981109)78:4<410::aid-ijc3>3.0.co;2-x

[bib28] Schafer JL (1999) Multiple imputation: a primer. Stat Methods Med Res 8: 3–15 10347857 10.1177/096228029900800102

[bib29] Sorkin JD, Muller DC, Andres R (1999) Longitudinal change in height of men and women: implications for interpretation of the body mass index. Am J Epidemiol 150: 969–977 10547143 10.1093/oxfordjournals.aje.a010106

[bib30] van den Brandt PA, Spiegelman D, Yaun SS, Adami HO, Beeson L, Folsom AR, Fraser G, Goldbohm RA, Graham S, Kushi L, Marshall JR, Miller AB, Rohan T, Smith-Warner SA, Speizer FE, Willett WC, Wolk A, Hunter DJ (2000) Pooled analysis of prospective cohort studies on height, weight, and breast cancer risk. Am J Epidemiol 152: 514–527 10997541 10.1093/aje/152.6.514

[bib31] Wadsworth MEJ, Butterworth S, Hardy R, Kuh D, Richards M, Langenberg C, Hilder WS, Connor M (in press) The life course prospective design; an example of benefits and problems associated with study longevity. Soc Sci Med10.1016/s0277-9536(03)00083-2 14512249

[bib32] Whitehead J, Carlile T, Kopecky KJ, Thompson DJ, Gilbert Jr FI, Present AJ, Threatt BA, Krook P, Hadaway E (1985) The relationship between Wolfe's classification of mammograms, accepted breast cancer risk factors, and the incidence of breast cancer. Am J Epidemiol 122: 994–1006 4061448 10.1093/oxfordjournals.aje.a114203

[bib33] Willett WC, Browne ML, Bain C, Lipnick RJ, Stampfer MJ, Rosner B, Colditz GA, Hennekens CH, Speizer FE (1985) Relative weight and risk of breast cancer among premenopausal women. Am J Epidemiol 122: 731–740 4050766 10.1093/oxfordjournals.aje.a114156

[bib34] Wolfe JN (1976) Breast patterns as an index of risk for developing breast cancer. Am J Roentgenol 126: 1130–1139 179369 10.2214/ajr.126.6.1130

